# Hodgkin Reed-Sternberg Cells of Classic Hodgkin Lymphoma: Morphology, Phenotype, Genotype, and Cell of Origin

**DOI:** 10.3390/cancers18091446

**Published:** 2026-04-30

**Authors:** Annunziata Gloghini, Daniele Lorenzini, Chiara Costanza Volpi, Desirè Viola Trupia, Giancarlo Pruneri

**Affiliations:** 1 Department of Advanced Diagnostics, Fondazione IRCCS, Istituto Tumori Milano, 20133 Milan, Italy; daniele.lorenzini@istitutotumori.mi.it (D.L.); chiara.volpi@istitutotumori.mi.it (C.C.V.); desire.trupia@istitutotumori.mi.it (D.V.T.); 2 Oncology and Hemato-Oncology Department, University of Milan, 20133 Milan, Italy

**Keywords:** Hodgkin Reed-Sternberg cells, classic Hodgkin lymphoma, morphology, phenotype, genotype, cell of origin

## Abstract

Classic Hodgkin lymphoma (cHL) is a distinctive B-cell malignancy defined by the presence of scarce but pathobiologically dominant Hodgkin Reed-Sternberg (HRS) cells within an inflammatory tumor microenvironment (TME). Although representing less than 10% of total tumor cellularity, HRS cells shape the TME by recruiting and functionally polarizing immune and stromal elements through cytokine- and chemokine-mediated signaling. Morphologically, HRS cells are large, atypical, often binucleated or multinucleated cells with prominent eosinophilic nucleoli and abundant cytoplasm, giving rise to the classic “owl’s eye” appearance. Distinct morphological variants—including lacunar, mummified, mononuclear, and anaplastic forms—contribute to the histopathologic diversity across cHL subtypes such as nodular sclerosis, mixed cellularity, lymphocyte-rich, and lymphocyte-depleted disease. The immunophenotype of HRS cells is equally characteristic, with strong and uniform CD30 expression, frequent CD15 positivity, reduced expression of B-cell markers (CD20, CD79A/B), and partial retention of PAX5, reflecting profound lineage dysregulation. Aberrant expression of activation markers and immune-evasion molecules, including PD-L1 driven by recurrent 9p24.1 amplification, underscores their capacity for immune escape. Genetically, HRS cells display alterations affecting *NF-κB*, *JAK/STAT*, and *PI3K/AKT* pathways, facilitated by somatic mutations, chromosomal gains, and epigenetic remodeling that silence B-cell-defining genes. Despite reprogramming, clonality and somatic hypermutation patterns confirm their origin from germinal center B-cells, even in EBV-associated cases. Collectively, the morphology, phenotype, and genotype of HRS cells reveal a complex pathogenic network in which intrinsic oncogenic pathways and extrinsic TME interactions co-operate to sustain malignant transformation. Understanding these integrated mechanisms provides a biological foundation for current therapeutic strategies.

## Simple Summary

Classic Hodgkin lymphoma (cHL) is a unique B-cell–derived malignancy characterized by rare but biologically dominant Hodgkin Reed–Sternberg (HRS) cells embedded within an inflammatory tumor microenvironment (TME). HRS cells profoundly remodel the TME through cytokine- and chemokine-driven recruitment and polarization of immune and stromal populations. Morphologically, HRS cells are large, atypical, and frequently multinucleated, with prominent eosinophilic nucleoli, and include diverse variants that contribute to the histologic spectrum of cHL subtypes. Their immunophenotype is defined by uniform CD30 expression, frequent CD15 positivity, diminished B-cell markers, and partial PAX5 retention, reflecting extensive lineage dysregulation. Aberrant signaling, including PD-L1 overexpression linked to 9p24.1 amplification, enables immune evasion. Genomic alterations affecting NF-κB, JAK/STAT, and PI3K/AKT pathways, together with epigenetic silencing of B-cell programs, further support malignant transformation. These integrated morphological, phenotypic, and genetic features form the basis for precise diagnosis and current therapeutic strategies in cHL.

## 1. Introduction

The classification of Hodgkin lymphoma (HL) has progressively evolved, driven by advances in histopathological evaluation, immunophenotypic characterization, and molecular diagnostic techniques. The 5th edition of the *WHO Classification of Lymphoid Tumors* [1] formalized the distinction between two major entities: classic Hodgkin lymphoma (cHL) and nodular lymphocyte predominant Hodgkin lymphoma (NLPHL). Classic HL represents approximately 95% of all HL cases, whereas NLPHL constitutes a separate clinicopathological entity with preserved B-cell features.

Classic HL represents a unique hematologic malignancy characterized by a minority of neoplastic Hodgkin Reed-Sternberg (HRS) cells embedded within a rich but non-neoplastic inflammatory microenvironment [2,3]. Despite usually accounting for less than 10% of the total cellularity, HRS cells orchestrate the recruitment, activation, and functional polarization of surrounding immune and stromal cells, creating a tumor-supportive niche essential for their survival [4,5]. This interaction between malignant and reactive elements is a defining biological and pathological hallmark of cHL and influences both clinical behavior and response to therapy.

Historically, cHL has been classified into four histologic subtypes—nodular sclerosis (NS), mixed cellularity (MC), lymphocyte-rich (LR), and lymphocyte-depleted (LD)—based on architectural patterns, morphology, and differences in inflammatory infiltrates [6,7,8]. Although these subtypes exhibit varying clinical presentations and epidemiologic associations, they all share a common dependence on the immunosuppressive tumor microenvironment (TME) shaped by HRS cells. Understanding the biology of HRS cells is therefore critical to unravel the pathogenesis of the disease.

Morphologically, HRS cells are large, atypical B-lineage cells with characteristic multinucleation, prominent nucleoli, and abundant cytoplasm [3,7]. Their immunophenotypic profile is equally distinctive, defined by strong CD30 expression, frequent CD15 positivity, reduced expression of classical B-cell markers, and aberrant expression of proteins associated with other hematopoietic lineages [3,7]. These features underscore a profound disruption of normal B-cell identity.

At the genetic level, HRS cells harbor recurrent somatic mutations and chromosomal alterations that drive deregulated signaling through pathways such as *NF-κB*, *JAK/STAT*, and *PI3K/AKT* [9,10,11]. Epigenetic silencing further contributes to lineage infidelity and immune evasion [12]. Despite these aberrations, molecular analyses confirm that HRS cells arise clonally from germinal center (GC) B-cells [13].

This review synthesizes current knowledge on the morphology, phenotype, genotype, and cell of origin of HRS cells, integrating these dimensions to highlight the biological variability across cHL subtypes.

## 2. Morphology of HRS Cells and Histologic Subtypes

The recognition of HRS cells by morphology remains a cornerstone for the diagnosis and classification of cHL. Typical HRS cells are large (approximately 20–60 μm) and display abundant amphophilic cytoplasm, often with a perinuclear clearing. Their nuclei are multinucleated or multilobated, containing finely dispersed chromatin and prominent eosinophilic nucleoli, which confer the characteristic “owl’s eye” appearance. In addition to these canonical features, a range of morphological variants contributes to the histopathological diversity of cHL (Table 1). These include mononuclear forms (Hodgkin cells), which share cytological features with HRS cells but contain a single nucleus; mummified cells, showing features consistent with apoptosis; lacunar cells, typically associated with the NS subtype; and anaplastic or pleomorphic variants, which may resemble cells seen in anaplastic large cell lymphoma, thereby posing challenges in differential diagnosis (Table 1).

These diagnostic cells typically reside within a polymorphous inflammatory background composed of small lymphocytes, eosinophils, plasma cells, histiocytes, and fibroblasts [7,8,14].

Morphologic diversity of HRS cells contributes to the distinction among the major histologic subtypes of cHL (Table 2). NS, the most prevalent subtype in Western populations, is defined by fibrous bands dividing the lymph node into nodules and by the presence of lacunar variant HRS cells. These cells display artifactual retraction of the cytoplasm, producing a perinuclear “lacuna” that becomes more apparent in formalin-fixed tissue. In high-grade variant of NS cHL, HRS cells can constitute dense aggregates with central necrosis (the so-called syncytial variant) [7,8,14,15].

**Figure 1:**
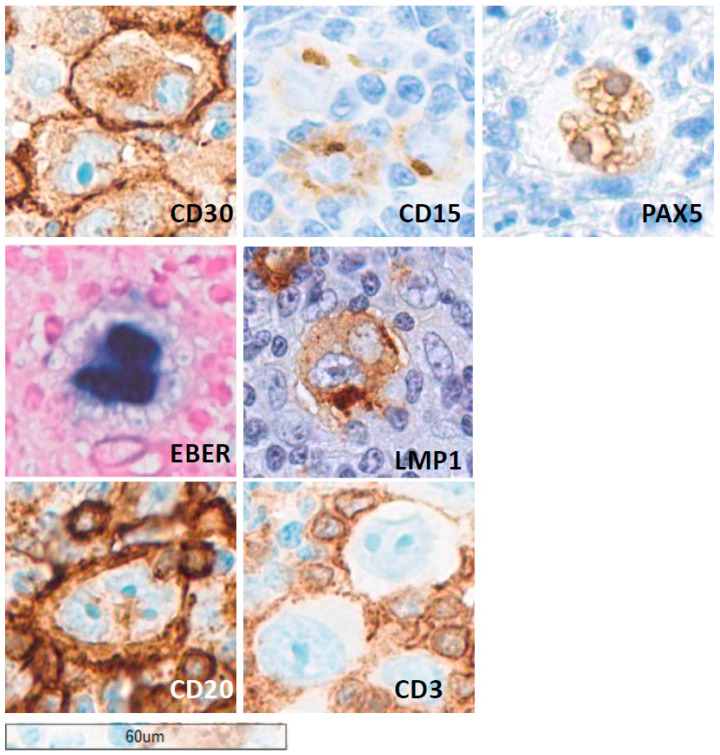
Immunophenotypic features of an example of mixed cellularity classic Hodgkin lymphoma (cHL). Immunohistochemistry demonstrates strong CD30 expression in HRS cells, a defining diagnostic feature, with CD15 positivity in the majority of cases and weak PAX5 expression consistent with B-cell derivation. Epstein–Barr virus (EBV) association is assessed by in situ hybridization for EBER and immunohistochemistry for LMP1, highlighting EBV-infected Hodgkin–Reed–Sternberg (HRS) cells. In a subset of cases, CD20 expression supports B-cell lineage. CD3 staining highlights surrounding T lymphocytes and may show rosetting around HRS cells, which should not be misinterpreted as true tumor cell positivity. Collectively, these features support the diagnosis of cHL and aid in its distinction from other CD30^+^ lymphoid proliferations. Scale bar, 60 μm.

Mixed cellularity cHL demonstrates a broader spectrum of inflammatory infiltrates and tends to contain classic, mononuclear, and binucleated HRS cells. This subtype has a stronger association with Epstein–Barr virus (EBV) (Figure 1), which influences both morphology and immunophenotype [16,17].

Lymphocyte-rich cHL, in contrast, is characterized by a relatively sparse number of HRS cells within a background dominated by small B lymphocytes and scattered histiocytes. In this subtype, HRS cells may be less pleomorphic and sometimes resemble centroblasts, complicating differential diagnosis with NLPHL, which cannot be reliably done without immunophenotypic characterization [2,7].

Lymphocyte depleted cHL represents the rarest and most aggressive variant, marked by numerous pleomorphic HRS cells, extensive necrosis, and a paucity of reactive lymphocytes [18]. In this setting, bizarre and anaplastic forms of HRS cells may predominate, mimicking high-grade non-Hodgkin lymphomas or even metastatic carcinoma.

Additional morphologic variants enrich the diagnostic landscape. Mummified cells exhibit nuclear pyknosis and shrunken eosinophilic cytoplasm, frequently appearing in advanced disease and fibrotic areas. Mononuclear Hodgkin cells are considered earlier developmental forms of classic HRS cells. Together, these morphologic features aid in distinguishing cHL subtypes and contribute to their clinical and biological heterogeneity.

## 3. Phenotype of HRS Cells

The immunophenotypic profile of HRS cells is central to their recognition and to differentiating cHL from morphologic mimics (Table 3, Figure 1). The most consistent and defining marker is CD30, a member of the TNF receptor superfamily that is uniformly expressed on HRS cells [7,19] and forms the basis for several targeted therapies, including brentuximab vedotin [14]. CD15 is present in approximately 75% of cases, at least in a fraction of HRS cells, though its absence does not exclude the diagnosis. Together, CD30 and CD15 form the classic immunophenotypic signature of cHL. Despite their confirmed B-cell derivation, HRS cells show a striking downregulation of typical B-cell markers. Expression of CD19, CD20, CD79A/B, and surface or cytoplasmic immunoglobulin is usually lost [20]. Along this line, CD45 expression is absent in almost all cHL cases; however, in some cases, its evaluation may be challenging, helping in differential diagnosis with other lymphomas [21]. The B-cell transcription factor PAX5 is consistently expressed but at reduced intensity, reflecting partial retention of B-lineage identity (Figure 1) [20]. Conversely, markers associated with activated lymphocytes and non-B-lineage cells, such as MUM1/IRF4, CD40, and TNF receptor family proteins, are frequently expressed [22,23,24]. This immunophenotypic aberrancy reflects profound transcriptional reprogramming rather than true multilineage differentiation. Transcription factors involved in B-cell activation and immunoglobulin gene expression—such as OCT2 and BOB1—are often either absent or weakly expressed. This feature contributes to the paradox of a B-cell-derived tumor lacking functional B-cell characteristics. HRS cells also frequently express proteins associated with immune evasion, including PD-L1, driven by 9p24.1 amplification or EBV infection [25]. This immunosuppressive phenotype facilitates escape from cytotoxic T-cell surveillance. The TME further shapes HRS cell immunophenotype through cytokine-driven signaling. Autocrine and paracrine loops involving IL-13, IL-21, and CD40L enhance the activated, survival-oriented phenotype characteristic of HRS biology [4]. Collectively, this distinctive immunophenotype, marked by CD30 expression, partial B-cell identity, and aberrant activation markers, remains central to diagnostic classification and therapeutic targeting.

## 4. Tumor Microenvironment and Cellular Composition Among cHL Subtypes

Classic HL presents a remarkable histopathological paradox: the malignant HRS cells constitute only a small fraction of the tumor mass—often less than 10%—while the majority comprises reactive and stromal cells. This unique configuration reflects the critical role of the TME in disease pathogenesis and progression. As mentioned above, the TME consists of stromal elements surrounded by a diverse infiltrate of non-neoplastic immune cells, including CD4^+^ and CD8^+^ T-cells, B-cells, eosinophils, mast cells and tumor-associated macrophages (TAMs). In cHL, TME is not a passive background but a dynamic and supportive cellular network that closely interacts with the malignant cells, shaping the disease’s clinical and biological behavior [4,14,26]. Recent advances in spatial and single-cell technologies have provided novel insights into the organization of the tumor microenvironment in cHL. In particular, the mononuclear phagocyte network—including monocytes, macrophages, and dendritic cells—displays marked spatial heterogeneity and functional diversity. These cells are not uniformly distributed but form structured niches in close proximity to HRS cells, where they may contribute to immune regulation, antigen presentation, and support of tumor cell survival. Spatial and molecular profiling studies have highlighted the complexity of these interactions, underscoring the role of myeloid populations as key components of the cHL microenvironment [27,28].

The composition of the TME distinctly varies among NS, MC, LR, and LD cHL subtypes (Table 2). Each subtype exhibits distinct histologic and immunologic patterns, reflecting differences in the cellular environment that supports it [29].

In NS cHL, the TME is dominated by fibroblasts and other stromal cells, producing dense fibrous bands that divide lymphoid tissue into nodules. This fibrotic framework is a hallmark feature of NS cHL, contributing to the architectural distortion observed in histological sections [7,8,29]. In contrast, MC cHL presents with a rich, polymorphic infiltrate of immune cells, including eosinophils, plasma cells, neutrophils, mast cells, and both B and T lymphocytes. These immune elements surround HRS cells and are recruited through a web of cytokines and chemokines secreted by the tumor cells themselves [7,8,29]. LD cHL, though rare, is characterized by a scarcity of small lymphocytes, an abundance of histiocytes and atypical HRS cells. The fibrotic component is irregular, and the overall TME is less organized than in NS cHL [7,8,18,29]. Meanwhile, LR cHL resembles NLPHL, since both demonstrate a lymphocyte-rich environment, although NLPHL is more often associated with follicular dendritic cells and organized nodular structures [7,8].

In addition to the well-characterized reactive T-cell infiltrate, several studies have investigated the T-cell receptor (TCR) repertoire in the tumor microenvironment of cHL. Recent high-throughput and single-cell analyses indicate that, despite the abundance of T-cells, the repertoire is largely diverse, with only limited clonal expansion, suggesting a constrained or ineffective antigen-driven response [30,31]. Consistently, molecular studies based on TCR gene rearrangement analysis have shown that clearly detectable clonal T-cell populations are relatively uncommon in cHL, further supporting the notion that T-cell clonality is not a dominant feature of the disease [32]. The biological significance of these findings remains incompletely understood. On the one hand, restricted or oligoclonal T-cell expansions may reflect antigen-driven responses to HRS cells or viral antigens, particularly in EBV-associated cases. On the other hand, the limited extent of clonal expansion suggests that the cHL microenvironment may actively impair effective T-cell priming and expansion. Overall, these observations highlight the complex and often dysfunctional interplay between HRS cells and the surrounding immune compartment.

### Tumor Microenvironment and Immune Evasion

Building on the compositional heterogeneity of TME, functional interactions between HRS cells and surrounding immune elements play a central role in immune evasion and disease persistence.

**Figure 2:**
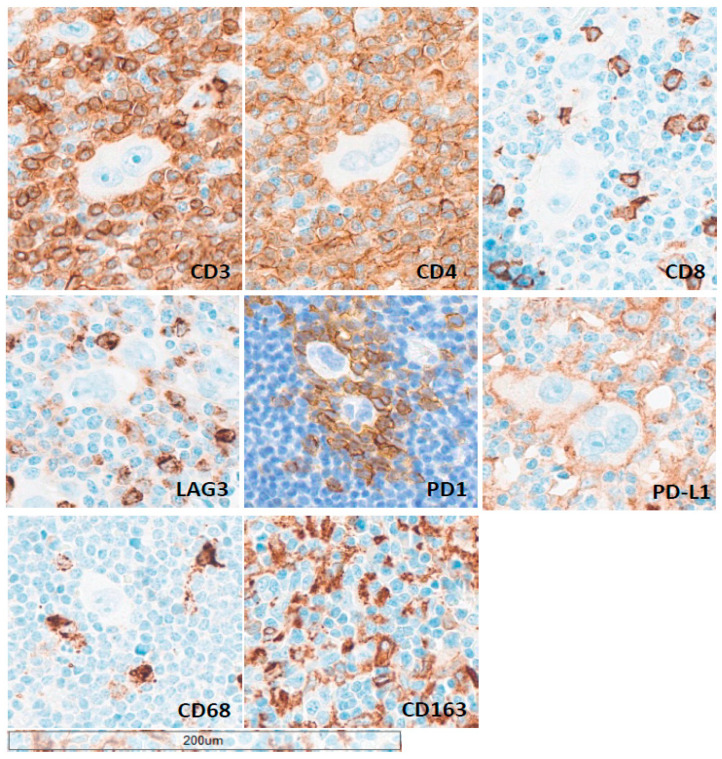
Spatial organization of exhausted T-cells and tumor-associated macrophages in the classic Hodgkin lymphoma (cHL) tumor microenvironment. Representative immunohistochemical staining of a nodular sclerosis cHL lymph node showing the cellular composition and spatial architecture of the tumor microenvironment. Panels illustrate T-cell populations (CD3, CD4, CD8) and expression of inhibitory receptors associated with T-cell exhaustion (PD-1, LAG-3), alongside PD-L1 expression within the microenvironment. Macrophage subsets are highlighted by CD68 and CD163 staining, with CD163^+^ tumor-associated macrophages (TAMs) representing an immunosuppressive M2-like phenotype. Tumor-infiltrating lymphocytes exhibit features of functional exhaustion, as indicated by checkpoint receptor expression, while TAMs contribute to an immunosuppressive niche. The spatial proximity of PD-1^+^ T-cells, PD-L1-expressing cells, and macrophages around Hodgkin–Reed–Sternberg cells supports the formation of PD-1/PD-L1-rich microenvironments that facilitate immune evasion. Scale bar, 200 μm.

Exhaustion and immune evasion in cHL are tightly linked to the cellular composition and spatial organization of the TME (Figure 2). TAMs, particularly CD163^+^ M2-like subsets, contribute to an immunosuppressive niche through cytokine secretion, impaired antigen presentation, and support of HRS cell survival [33,34,35]. Concurrently, tumor-infiltrating T-cells exhibit exhaustion programs characterized by sustained expression of inhibitory receptors such as PD-1, LAG-3, TIM-3, and TIGIT, reflecting progressive loss of effector function [5,36]. Co-expression of these checkpoints identifies highly dysfunctional T-cell populations and provides a mechanistic basis for immune escape. Spatial analyses further demonstrate that suppressive immune cells cluster in close proximity to HRS cells, forming PD-1/PD-L1-rich niches that reinforce T-cell exhaustion [37,38].

## 5. Functional Expression of Key Markers on Tumor Tissue and cHL Tumor Cell Lines

HRS cells constitute only a minor fraction of the tumor mass. Notably, their survival and proliferation depend on a complex interplay between tumor-intrinsic oncogenic pathways and signals derived from the TME. Functional analyses of tumor biopsies and HRS-derived cell lines have clarified the molecular networks that sustain this disease, revealing prominent roles for CD30, CD40, PD-L1, IRF4, and NF-κB in sustaining cell survival, immune evasion, and cytokine-driven inflammation. HRS cell lines such as L428, KM-H2, and L1236 allowed researchers to dissect receptor-mediated signaling, transcriptional regulation, and cytokine responses in controlled conditions [24,39]. These models have directly informed therapeutic innovation, particularly the development of CD30-directed antibody–drug conjugates and PD-1/PD-L1 checkpoint inhibitors, which represent cornerstones of modern cHL therapy [14,40]. The following sections summarize key functional properties of major molecular pathways relevant to HRS biology, integrating evidence from tumor tissue and in vitro studies.

### 5.1. CD30’s Expression and Function in HRS Cells

CD30, a member of the tumor necrosis factor receptor (TNFR) superfamily, is among the most consistent markers of HRS cells, with minimal expression in resting lymphoid cells [19,41,42]. In HRS-derived cell lines, CD30 ligation activates NF-κB and MAPK pathways, enhancing survival, cytokine secretion, and proliferation [43,44]. Functional assays demonstrate that CD30 cross-linking promotes activation of AP-1 and NF-κB transcription factors, protecting HRS cells from apoptosis even in the absence of B-cell receptor signaling [43,44].

#### 5.1.1. CD30’s Role in Modulating the Tumor Microenvironment

CD30 substantially contributes to TME remodeling. Through interactions with CD30 ligand (CD30L, expressed on bystander immune cells), CD30 signaling induces the secretion of pro-inflammatory cytokines that recruit regulatory T-cells and suppress cytotoxic T-cell responses. Soluble CD30, shed from HRS surfaces, can further dampen T-cell activity and promote immune evasion [19,41,42].

#### 5.1.2. CD30’s Therapeutic Implications

CD30’s restricted expression pattern has enabled effective targeted therapy. Brentuximab vedotin, a CD30-directed antibody–drug conjugate, selectively delivers cytotoxic payloads to HRS cells, significantly improving outcomes in relapsed/refractory cHL, and is currently recommended both in relapsed/refractory cHL and in first-line settings [45,46,47,48].

### 5.2. PD-L1’s Expression and Function in HRS Cells

PD-L1 (CD274), a key immune checkpoint regulator, is highly expressed in HRS cells owing largely to recurrent 9p24.1 amplification, which simultaneously increases PD-L1, PD-L2, and JAK2 copy numbers [11,25]. Cell line studies confirm that IFN-γ exposure further augments PD-L1 expression, generating a feedback loop that reinforces immune suppression [11,25]. PD-L1 on HRS cells binds PD-1 receptors on cytotoxic T lymphocytes, inhibiting their activation and fostering T-cell exhaustion. This mechanism is central to tumor immune evasion [40,49,50].

#### 5.2.1. PD-L1’s Contribution to the Tumor Microenvironment

Beyond tumor cells, PD-L1 is abundantly expressed on tumor-associated macrophages (TAMs) [51] (Figure 1), dendritic cells, and other stromal components in the TME [33]. These PD-L1^+^ cells cluster around HRS cells, establishing a spatially organized immunosuppressive niche that impairs antitumor immunity [27,52,53,54,55].

#### 5.2.2. PD-L1’s Therapeutic Implications

Checkpoint inhibitors targeting PD-1—such as nivolumab and pembrolizumab—have demonstrated robust efficacy in heavily pretreated cHL patients. Their success underscores the central role of the PD-1/PD-L1 axis in sustaining the immune-permissive environment characteristic of cHL [40,49,50,56].

### 5.3. CD40’s Expression and Intrinsic Functional Roles

**Figure 3:**
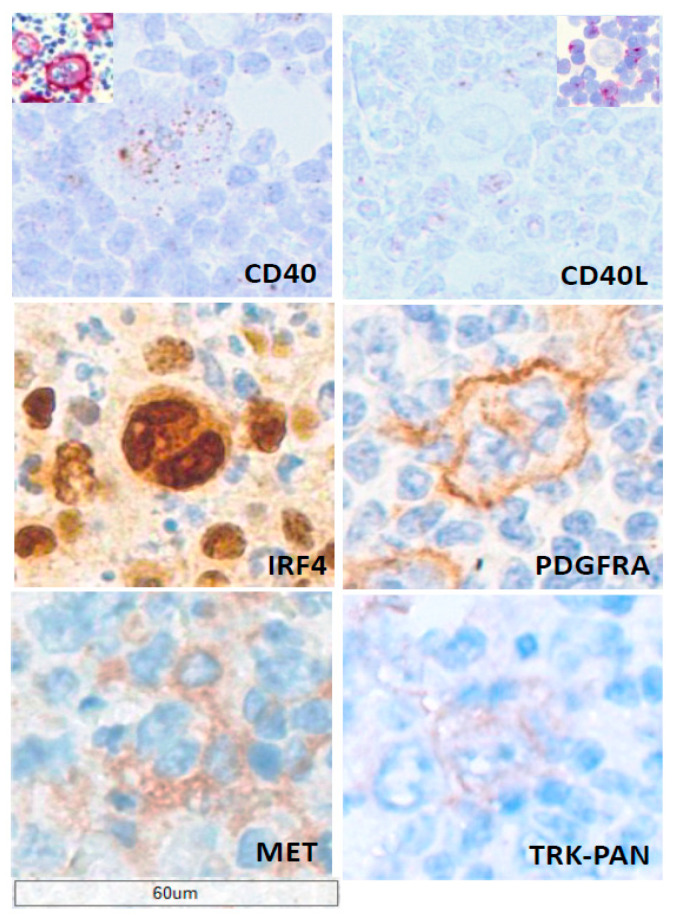
Expression of CD40/CD40L axis, IRF4, and receptor tyrosine kinases in nodular sclerosis classical Hodgkin lymphoma (cHL). Representative staining of cHL tissue illustrating key signaling pathways in Hodgkin and Reed–Sternberg (HRS) cells. High CD40 mRNA expression, as demonstrated by in situ hybridization (ISH), leads to strong CD40 protein expression in HRS cells (inset). CD40L (CD154) is detected by mRNA ISH and immunocytochemistry (inset, cell suspension) in surrounding lymphocytes that provide extrinsic stimulation to HRS cells. Engagement of CD40 by CD40L promotes activation of NF-κB and MAPK pathways, supporting HRS cell survival, proliferation, and resistance to apoptosis. Nuclear expression of IRF4 is evident in HRS cells, consistent with its role in regulating genes involved in survival, proliferation, and immune signaling. In addition, HRS cells express receptor tyrosine kinases (RTKs), including PDGFRA and c-MET, as well as TRK family members, reflecting activation of the *PI3K*/*AKT*/*mTOR* and *MAPK* signaling pathways. Collectively, these pathways contribute to proliferation, adhesion, and resistance to cell death and may be driven by autocrine or paracrine ligand interactions within the tumor microenvironment. Scale bar, 60 μm.

CD40, another TNFR family receptor, is highly expressed on HRS cells (Figure 3) [24]. Engagement by CD40L potently activates NF-κB and MAPK pathways, promoting survival, proliferation, and resistance to apoptosis [57,58]. In vitro stimulation of cHL cell lines increases anti-apoptotic proteins such as BCL-XL and boosts the secretion of IL-6 and IL-13, which contribute to the inflammatory milieu [58]. CD40 signaling also maintains partial B-cell lineage identity in HRS cells by inducing transcription factors such as BCL6 and IRF4, despite the loss of a functional B-cell receptor.

#### 5.3.1. CD40’s Impact on the Tumor Microenvironment

The CD40–CD40L axis contributes to immune cell recruitment and TME structuring. CD40 activation induces secretion of chemokines that attract T-helper cells, monocytes, and regulatory T-cells, reinforcing chronic inflammation and tumor support. CD40 is also found on certain stromal and myeloid cells, suggesting reciprocal signaling loops that strengthen tumor–stroma interactions [58].

#### 5.3.2. CD40’s Therapeutic Potential

Although CD40-targeted agents are not yet approved for cHL, agonistic antibodies and pathway modulators under investigation in other lymphomas may hold promise for disrupting survival signaling in HRS cells [42,58].

### 5.4. IRF4’s Expression and Role in HRS Cells

IRF4, a transcription factor essential for lymphoid differentiation and plasma cell development, is strongly expressed in HRS cells (Figure 3) [22,23]. Functional studies using HRS cell lines demonstrate that IRF4 regulates survival, proliferation, and immune signaling by controlling genes such as c-MYC and components of the NF-κB network [59,60,61].

#### 5.4.1. IRF4’s Modulation of the Tumor Microenvironment

Through its transcriptional activity, IRF4 influences cytokine and chemokine production, particularly IL-6 and IL-21, which promote T-helper recruitment and plasma cell differentiation. These immune-modulating effects indirectly reinforce the tumor-supportive microenvironment [59,61,62].

#### 5.4.2. IRF4’s Therapeutic Perspectives

Although no direct IRF4 inhibitors exist, targeting its downstream pathways (e.g., NF-κB, c-MYC) may provide indirect therapeutic opportunities.

### 5.5. CD40L’s (CD154) Role in HRS Cell Survival and Activation

CD40L, expressed primarily by activated CD4^+^ T-cells (Figure 3), is a crucial extrinsic activator of CD40 signaling in HRS cells. Co-culture experiments show that CD40L-expressing T-cells enhance the survival and proliferation of HRS cell lines, compensating for the absence of B-cell receptor signaling [24,58,60].

#### 5.5.1. CD40L’s (CD154) Impact on the Microenvironment

CD40L-mediated activation increases secretion of inflammatory cytokines (IL-13, TNF-α) and chemokines (CCL5), which recruit additional immune cells to the tumor bed, sustaining chronic inflammation and tumor growth [4].

#### 5.5.2. CD40L’s (CD154) Therapeutic Considerations

Although CD40L itself is not a direct target, modulation of CD40–CD40L interactions may represent a strategy for reprogramming the TME and weakening tumor–immune crosstalk [63].

### 5.6. NF-κB’s Constitutive Activation in HRS Cells

NF-κB is a master transcription factor governing inflammation, immunity, and cell survival. In cHL, its constitutive activation is a defining pathogenic feature [44,64]. Both primary tumor samples and HRS-derived cell lines exhibit nuclear localization of NF-κB subunits, indicating persistent activation [44,64]. This activation results from genetic lesions (e.g., A20/TNFAIP3 mutations), external stimuli (e.g., CD40L), and EBV-derived proteins like LMP1, which mimic CD40 signaling [41,64,65].

#### 5.6.1. NF-κB’s Functional Consequences

NF-κB drives expression of anti-apoptotic proteins (BCL-XL), cytokines (IL-6, IL-13, TNF-α), and immune modulators including PD-L1 [11,44,64]. Inhibition of NF-κB results in rapid apoptosis in HRS cell lines, highlighting its essential role in tumor maintenance [44].

#### 5.6.2. NF-κB’s Influence on the Tumor Microenvironment

NF-κB-dependent chemokines (CCL5, CCL17) shape the characteristic immune infiltrate of cHL, perpetuating the tumor-supportive microenvironment [66].

#### 5.6.3. NF-κB’s Therapeutic Implications

Although direct NF-κB inhibition is challenging due to systemic toxicity, upstream targeting (CD30, CD40) or restoration of negative regulators such as A20 holds therapeutic promise [42].

## 6. Functional Integration and Pathogenic Network Overview

The signaling molecules described above operate as an interconnected network that sustains the malignant phenotype of HRS cells while shaping the TME to favor tumor survival. CD30 and CD40 activate NF-κB and IRF4, which orchestrate transcriptional programs that promote proliferation, immune evasion, and cytokine-driven inflammation. PD-L1, upregulated through NF-κB activity and chromosomal amplification, suppresses cytotoxic T-cell responses. IRF4 reinforces tumor identity and suppresses classical B-cell lineage programs. CD40L from T-helper cells establishes a critical extrinsic stimulus that maintains HRS viability and supports TME structuring. This feedback-rich signaling circuitry has not only deepened the understanding of cHL biology but also informed the development of targeted therapies, including PD-1 inhibitors and CD30-directed agents. Ongoing research into synergistic targeting of these pathways may yield future therapeutic advances [4,63,67].

Table 4 summarizes the main functional characteristics of key molecules expressed in tumor tissue and cHL cell lines.

## 7. Genotype of HRS Cells

HRS cells possess a complex and heterogeneous genetic landscape characterized by recurrent chromosomal gains, somatic mutations, and epigenetic alterations. These features promote proliferation, survival, and immune escape (Table 5) [68]. One of the most consistent genetic events is 9p24.1 amplification, which increases the dosage of PD-L1 and PD-L2, thereby activating immune checkpoint pathways. This cytogenetic alteration provides the rationale for checkpoint inhibitor therapy in relapsed and refractory cHL [11,25,40,69,70].

Recent studies have further refined the genomic landscape of cHL, suggesting the existence of biologically distinct subgroups characterized by different patterns of genetic alterations. Recurrent lesions affecting key signaling pathways, including *NF-κB*, *JAK/STAT*, and *PI3K*, are well documented and contribute to the constitutive activation of survival and proliferation programs in HRS cells. In addition, alterations involving genes such as *TP53* and epigenetic regulators (e.g., *KMT2D*) have been reported in subsets of cases. Emerging data from integrative and noninvasive genomic profiling approaches support the concept of molecular heterogeneity in cHL, although the definition of discrete genomic subtypes and their clinical implications are still being refined [14,71].

Disruption of the *NF-κB* pathway represents a central genetic hallmark. Inactivating mutations in *TNFAIP3* (A20) and alterations in *NFKBIA*, *NFKBIE*, and *REL* lead to constitutive pathway activation, preventing apoptosis and promoting cytokine expression [68]. EBV-positive cHL acquires NF-κB activation through latent membrane protein 1 (LMP1), which mimics CD40 signaling [65,69,72].

Parallel abnormalities affect the *JAK*/*STAT* pathway, with recurrent mutations in *SOCS1*, *STAT6*, and *JAK2* supporting uncontrolled proliferative signaling. Autocrine stimulation by IL-13 and IL-21 further amplifies this dysregulation. The emerging STAT3–BATF3–MYC transcriptional axis has been identified as a major driver of oncogenic transcription in HRS cells [68].

Alterations in the *PI3K*/*AKT*/*mTOR* pathway result from aberrant expression or activation of receptor tyrosine kinases (RTKs), including PDGFRA, DDR2, and TRKA/B (Figure 3), thereby promoting cell survival and metabolic adaptation [42]. The hepatocyte growth factor (HGF) receptor, c-Met, is an RTK expressed by HRS cells in the majority of cHL cases [73,74]. HGF expression by CD21^+^ dendritic reticulum cells and, in approximately 20% of cases, also by HRS cells, supports both paracrine and autocrine activation of c-Met-positive HRS cells [73,74].

These molecular features overlap with those observed in other mediastinal B-cell lymphomas, including primary mediastinal large B-cell lymphoma and mediastinal gray zone lymphoma, supporting the existence of a biological continuum among these entities, as highlighted by recent genomic and clinical studies [75,76].

Beyond genetic mutations, HRS cells exhibit widespread epigenetic reprogramming. Promoter hypermethylation and chromatin remodeling suppress expression of B-cell-defining genes, while transcriptional repressors including ID2 and ABF1 contribute to lineage infidelity. These epigenetic modifications help explain the loss of immunoglobulin expression and the paradoxical survival of GC B-cells with non-functional Ig genes—cells that would otherwise undergo apoptosis [68]. Together, the genetic and epigenetic architecture of HRS cells underpins their malignant phenotype and offers critical therapeutic targets.

Noninvasive genomic profiling using circulating tumor DNA (ctDNA) has emerged as a promising tool in cHL. Quantitative and qualitative analyses of ctDNA can reflect tumor burden and provide real-time insights into disease dynamics. Several studies have demonstrated the potential utility of ctDNA for monitoring treatment response and for the detection of minimal residual disease (MRD), with possible implications for early identification of relapse. While these approaches are not yet fully standardized for routine clinical use, they represent a rapidly evolving area with significant translational relevance [71,77].

## 8. Cell of Origin of HRS Cells

The cell of origin of HRS cells is well established as a GC-derived B-cell that has lost its typical B-cell phenotype (Table 6). In light of this consolidated knowledge, this aspect has been presented concisely. Conversely, the section addressing the genotype of HRS cells has been updated to incorporate recent findings, including recurrent genetic alterations and pathway dysregulation identified through next-generation sequencing approaches [12,13,71,78,79,80].

The survival of such aberrant B-cells is made possible by constitutive activation of prosurvival pathways, such as *NF-κB* and *JAK/STAT*, as well as by signals originating from the TME. This combination rescues cells that would normally undergo negative selection and allows them to undergo malignant transformation. Epigenetic repression of the B-cell program further masks their lineage affiliation, resulting in the characteristic loss of surface B-cell markers [10].

Although B-cell derivation is the rule, rare cases of cHL exhibit T-cell or uncommitted lymphoid precursor origins. These atypical cases, however, remain exceptional and do not alter the broader classification of cHL as a B-cell malignancy and should be considered in the differential diagnosis of a real T-cell lymphoma. EBV infection plays a prominent role in subsets of cHL, particularly MC and LD subtypes, where the virus contributes both to survival signaling and to the rewiring of cellular transcriptional programs [16,17]. EBV-positive HRS cells still originate from GC B-cells but acquire additional transcriptional and phenotypic properties imposed by viral proteins [81].

Overall, the GC B-cell origin of HRS cells provides a unifying biological framework across cHL subtypes. Recognizing this lineage supports accurate diagnosis and informs the interpretation of immunophenotypic abnormalities while also guiding efforts to understand lineage infidelity and survival mechanisms in malignant B-cells.

## 9. Conclusions

HRS cells exhibit remarkable variability in morphology, phenotype, and genotype, reflecting the biological complexity of cHL. Morphologically, these cells range from classic binucleated forms with prominent nucleoli to lacunar, mummified, and pleomorphic variants associated with specific histologic subtypes. Phenotypically, HRS cells maintain partial B-cell identity while simultaneously expressing a constellation of activation markers, immune checkpoint ligands, and lineage-incongruent proteins, underscoring extensive transcriptional dysregulation.

Genetically, HRS cells harbor diverse somatic mutations, chromosomal gains, and epigenetic modifications affecting pathways such as *NF-κB*, *JAK/STAT*, and *PI3K/AKT*. These alterations not only drive malignant transformation but also shape the interaction between HRS cells and the TME. Despite this variability, the unifying origin of HRS cells from GC B lymphocytes anchors the disease within B-cell biology while highlighting the unique mechanisms through which these cells evade normal lineage constraints.

Future perspectives include the integration of noninvasive genomic profiling, such as circulating tumor DNA, advanced spatial transcriptomic approaches to dissect tumor–microenvironment interactions, and the development of combinatorial therapeutic strategies targeting both tumor-intrinsic pathways and the immune microenvironment. In addition, epigenetic-targeted therapies may further expand the therapeutic landscape of cHL.

## Figures and Tables

**Table 1:** Morphologic variants of HRS cells.

Variant	Morphologic Features	Associated Subtypes
Lacunar cells	Perinuclear cytoplasmic retraction artifact	Nodular sclerosis
Mummified cells	Nuclear pyknosis and cytoplasmic shrinkage	Advanced disease, lymphocyte depletion
Mononuclear Hodgkin cells	Single large nucleus; precursor of classic HRS cells	All subtypes
Pleomorphic cells	Highly atypical forms	Lymphocyte depletion

**Table 2:** Histologic subtypes of classic Hodgkin lymphoma *.

Subtype	Key Morphological Features	Inflammatory Background *	EBV Association	Clinical Notes
Nodular sclerosis (NS)	Lacunar HRS cells; broad fibrous bands	Mixed, often with eosinophils	Low	Most common in young adults
Mixed cellularity (MC)	Classic HRS cells; mononuclear forms	Dense, polymorphous	High	More common in older adults and developing regions
Lymphocyte-rich (LR)	Scant HRS cells; centroblast-like variants	Mixed, with B lymphocytes and histiocytes	Variable	Favorable prognosis
Lymphocyte-depleted (LD)	Numerous pleomorphic HRS cells; necrosis	Sparse lymphocytes	High	Rare, aggressive

* NS cHL represents the most frequent subtype and is characterized by collagenous fibrous bands that subdivide the lymphoid tissue into nodular structures. Lacunar-type HRS cells are typically prominent, and the background commonly includes eosinophils, plasma cells, and neutrophils. In contrast, MC cHL exhibits a diffuse growth pattern with numerous classic HRS cells within a heterogeneous inflammatory milieu composed of eosinophils, histiocytes, plasma cells, and small lymphocytes. Unlike NS cHL, fibrotic bands are absent in this subtype, which is frequently associated with Epstein–Barr virus infection. LR cHL is distinguished by relatively sparse HRS cells dispersed within a background predominantly composed of small B lymphocytes.

**Table 3:** Key immunophenotypic markers of HRS cells.

Marker	Expression Pattern	Diagnostic Utility
CD30	Strong, uniform	Essential diagnostic marker; therapeutic target
CD15	Positive in ~75%	Supportive but not essential
CD20/CD79A/B	Usually absent	Helps exclude B-cell lymphomas
PAX5	Weak	Confirms B-cell derivation
MUM1/IRF4	Positive	Indicates activation phenotype
PD-L1	Overexpressed	Immune evasion; target for checkpoint inhibitors

**Table 4:** Functional observations on tumor tissue and cHL cell lines.

Molecule	Expression in HRS Cells	Key Functions	Microenvironmental Effects	Therapeutic Relevance
CD30	Highly expressed	Activates NF-κB, MAPK; promotes survival	Regulates cytokine release; recruits Tregs; suppresses CTLs	Target of brentuximab vedotin
PD-L1	Overexpressed due to 9p24.1 amplification	Inhibits CTLs via PD-1 engagement	Promotes T-cell exhaustion; abundant on TAMs	Target of PD-1 inhibitors
CD40	Strongly expressed	Activates NF-κB/MAPK; induces anti-apoptotic proteins	Recruits T-helper cells, monocytes	Target under investigation
IRF4	Strongly expressed	Regulates c-MYC, NF-κB; supports survival	Controls cytokine output (IL-6, IL-21)	Indirect targeting possible
CD40L	Absent in HRS cells; highly expressed on activated CD4^+^ T-cells	Paracrine CD40 activation; enhances HRS survival	Induces chemokines recruiting T-cells, macrophages	Indirectly targetable through CD40 pathway
NF-κB	Constitutively active	Master regulator of survival and inflammation	Controls chemokine-driven immune infiltration	Targeting upstream pathways

**Table 5:** Major genetic and epigenetic alterations in HRS cells *.

Pathway	Key Abnormalities	Functional Impact
*NF-κB*	*TNFAIP3* mutations; *REL* amplifications	Constitutive survival signaling
*JAK*/*STAT*	*SOCS1*, *STAT6*, *JAK2* mutations	Proliferation, anti-apoptotic signaling
Immune evasion	9p24.1 amplification	PD-L1/PD-L2 upregulation
Antigen presentation	*B2M* mutations	Loss of MHC class I
Epigenetic regulation	Promoter hypermethylation; PRC activity	Loss of B-cell identity

* 9p24.1 amplification driving PD-L1/PD-L2 expression. *NF-κB* pathway alterations: *TNFAIP3* mutations, *REL* amplifications. *JAK/STAT* dysregulation: *SOCS1*, *STAT6*, and *JAK2* mutations. Activation of *PI3K*/*AKT*/*mTOR* via receptor tyrosine kinase upregulation. Epigenetic silencing of B-cell genes via hypermethylation and chromatin remodeling.

**Table 6:** Evidence supporting B-cell origin of HRS cells.

Evidence Type	Findings
Immunogenetics	Clonal Ig rearrangements, somatic hypermutation
Lineage markers	Weak PAX5, partial B-cell program
Survival pathways	NF-κB rescuing crippled GC B-cells
Rare exceptions	Occasional T-cell or uncommitted precursor origin

## Data Availability

No new data were created or analyzed in this study.
